# Strategies to develop antivirals against enterovirus 71

**DOI:** 10.1186/1743-422X-10-28

**Published:** 2013-01-22

**Authors:** Rei-Lin Kuo, Shin-Ru Shih

**Affiliations:** 1 Research Center for Emerging Viral Infections, Chang Gung University, 259 Wen-Hua 1st Road, Kwei-Shan, Taoyuan, Taiwan; 2 Department of Medical Biotechnology and Laboratory Science, College of Medicine, Chang Gung University, Taoyuan, Taiwan; 3 Institute of Biotechnology and Pharmaceutical Research, National Health Research Institutes, Miaoli, Taiwan

## Abstract

Enterovirus 71 (EV71) is an important human pathogen which may cause severe neurological complications and death in children. The virus caused several outbreaks in the Asia-Pacific region during the past two decades and has been considered a significant public health problem in the post-poliovirus eradication era. Unlike poliovirus, there is no effective vaccine or approved antivirals against EV71. To explore anti-EV71 agents therefore is of vital importance. Several strategies have been employed to develop antivirals based on the molecular characteristics of the virus. Among these, some small molecules that were developed against human rhinoviruses and poliovirus are under evaluation. In this review, we discuss the recent development of such small molecules against EV71, known drug resistance and possible solutions to it, and animal models for evaluating the efficacy of these antivirals. Although further investigation is required for clinical applications of the existing candidates, the molecular mechanisms revealed for the inhibition of EV71 replication can be used for designing new molecules against this virus in the future.

## Introduction

Enterovirus 71 (EV71) belongs to the genus *Enterovirus* of the family *Picornaviridae*. Since the virus was first isolated in California in 1969 [1], a number of EV71 outbreaks have been reported throughout the world, from North America and Europe to Australia and Asia. During the past two decades, the virus has seriously affected the Asia-Pacific area, becoming a public health concern in several countries within this area [2-5]. Most commonly, EV71 causes hand-foot-and-mouth disease (HFMD) in children, which is considered a mild syndrome. However, some young children infected by the virus have developed severe neurological syndromes, such as aseptic meningitis, encephalitis, poliomyelitis-like paralysis, and even death [6]. For example, the 1998 outbreak in Taiwan resulted in 405 cases of severe neurological complications, pulmonary edema or hemorrhage, and myocarditis, with 78 deaths [3]. More recently, a large scale EV71 outbreak associated with HFMD, neurological symdrome, and fatal cases was reported in China [5,7]. Until 2012, fatal cases caused by EV71 infection were still described in Asia [8].

Similar to other human enteroviruses, such as poliovirus, the transmission of EV71 occurs through the fecal-oral route. The primary replication sites for the virus are presumed to be in the tonsils and intestinal lymphoid tissue. Therefore, the virus can be spread from the gastrointestinal tract of infected people for weeks. This may also explain why most EV71 isolates were recovered from throat swabs and stool specimens. However, clinical features have suggested that the virus can reach the central nervous system (CNS) of people suffering from EV71 infection [5,9]. The two explanations for how EV71 spreads to the CNS are that virus in the bloodstream somehow penetrates the blood-brain barrier, or that virus reaches the CNS via a neuronal route. Recently, a mouse study supported the proposition that the major transmission route to the CNS may be through retrograde axonal transport in neurons [10].

In past decades, in an effort to eradicate polio, a dramatic reduction of epidemics has occurred through effective vaccines and improvement in public hygiene. However, the emergence of EV71 infection has recently developed into a new threat to children, especially because there are no specific treatments or vaccines to combat this ailment. Supportive therapy is still the primary management for severe cases of EV71 infection. Other than symptomatic treatment, intravenous immunoglobulin (IVIG) is clinically used to neutralize the virus and to nonspecifically suppress inflammation. Nevertheless, although this treatment has been routinely applied to severe cases of EV71 infection, its efficacy requires further evaluation. Therefore, the development of specific antiviral strategies against EV71 has become an urgent issue for the protection of children from the hazards of EV71 infection.

### Biological characteristics of EV71

Like poliovirus, the prototype virus in the family *Picornaviridae*, EV71 is a small non-enveloped virus that encloses a positive-sense, single-stranded RNA molecule of approximately 7.4 kilobases [11]. After initial infection, the virus particle attaches itself to receptors on host cells and releases its RNA genome into the cytoplasm. Two host proteins, scavenger receptor B2 and human P-selectin glycoprotein ligand-1, have been identified as cellular receptors for EV71 [12,13]. Once the genome enters the host cell, the viral RNA, which possesses an internal ribosomal entry site (IRES) and poly(A) tail, serves as mRNA and is translated by a cap-independent mechanism [14]. A single large polyprotein is first synthesized. The functional viral proteins are then generated through maturation cleavage mediated by viral proteases 2A^pro^ and 3C^pro^[15].

The viral RNA genome is not only the mRNA for viral protein translation, but it can also be the template for replication by the virus-encoded RNA-dependent RNA polymerase (RdRP), designated 3D. In the infected cell, viral RNA replication occurs within a vesicular membrane structure in the cytoplasm [16,17]. Other than the 3D protein, the viral 2C protein, which is highly conserved among human enteroviruses, has been identified as part of the viral replication complex within the vesicular membrane [17]. Thereafter, the progeny positive-stranded viral RNA is then packaged by viral capsid proteins to form a new infectious virion. Infection eventually initiates the apoptotic pathway by its 2A^pro^ and 3C^pro^ proteins and lyses the infected cell [18,19]. Newly produced viral particles are released from the lysed cell.

### Antivirals targeting virus entry

Blocking virus entry is an ideal antiviral strategy. For example, to a certain extent, treating severe cases of EV71 infection with IVIG was partly dependent on its nonspecific neutralization of the virus [20]. Correspondingly, certain animal studies have shown that passive transfer of antiserum from immunized mice could provide protection against an EV71 challenge [21,22]. According to past research, among viral capsid proteins, neutralizing epitopes were located on the VP1 protein [23]. VP1 was also identified as the major receptor-binding protein after EV71 infection [12,13]. Thus, development of specific antibodies against the neutralizing epitope on the viral capsid protein VP1 could be a successful antiviral strategy.

**Figure 1:**
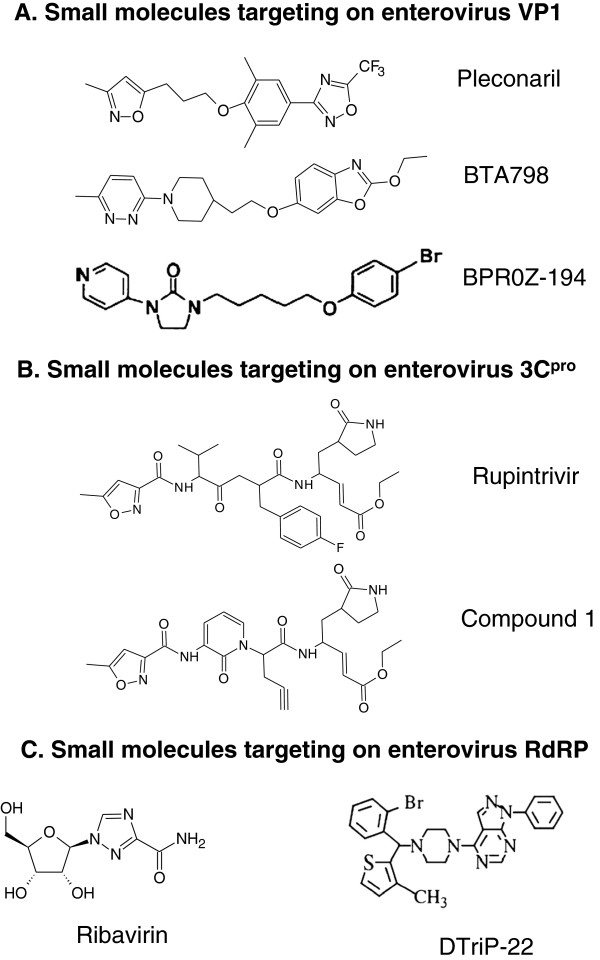
**Structures of small molecules targeting EV71 proteins.** We thank Dr. Johan Neyts for providing the structures of Pleconaril, BTA798, Rupintrivir, Compound 1, and Ribavirin.

The VP1 protein of poliovirus or rhinovirus has also been shown to form a canyon structure, which is important for receptor binding. In addition, conformational change of the VP1 protein is the crucial step for viral particle disassembly and release of viral RNA into the host cell. Therefore, designing small molecules that target VP1 may interfere with EV71 infection. Two molecules, pleconaril (Figure 1A) and pirodavir, were found to suppress rhinovirus replication by targeting the capsid protein [24-26]. One of the molecules, pleconaril, has already been applied to treat life-threatening enterovirus infections [27]. To verify the specific efficacy of pleconaril against EV71, a mouse study was performed, which resulted in significant improvement of severe symptoms in infected 1-day-old mice [28]. However, the EV71 inhibition capacity of pleconaril could vary for different isolates of the virus. Shia et al. reported that pleconaril was nearly ineffective in neutralizing EV71 isolates from the outbreak in Taiwan [29].

By using the skeleton of pleconaril as a template, a computer-assisted drug design developed a new class of pyridyl imidazolidinones with anti-EV71 activity. Among the pyridyl imidazolidinones, one of the compounds, BPR0Z-194 (Figure 1A), demonstrated its effectiveness against EV71 replication [30]. In addition to VP1-binding small molecules, lactoferrin, which is an abundant iron-binding glycoprotein in colostrum, was found to inhibit EV71 by binding to VP1 [31]. Although lactoferrin has not been approved for therapeutic purposes, it could be considered an agent for preventing virus entry.

### Antivirals targeting enterovirus proteases 3C^pro^ and 2A^pro^

Maturation cleavages are critical steps for EV71 protein synthesis. As for other human enteroviruses, 2A^pro^ and 3C^pro^ are the key proteases for processing of the viral precursor polyprotein. Rupintrivir (Figure 1B) was originally developed to inhibit human rhinovirus infection by blocking its 3C^pro^ activity [32]. In recent years, this compound was confirmed as an EV71 replication inhibitor through the blockage of its 3C^pro^ activity [33-35]. In addition to rupintrivir, Compound 1 (Figure 1B), which has been shown to inhibit the human rhinovirus 3C protease, may be another candidate for EV71 treatment by suppressing EV71 3C^pro^[36]. To further investigate the antagonism of 3C^pro^, structure-based design could be highly practical, especially because the X-ray crystal structure of the EV71 3C^pro^ has recently been resolved [37].

2A^pro^, which cleaves both the viral polyprotein and translational factor eIF4GI for shutting off host-cell translation, is also considered an antiviral drug target for inhibiting EV71 replication. Unfortunately, no specific inhibitor has been developed for blocking this chymotrypsin-related protease. Nevertheless, the X-ray crystal structures of 2A^pro^ from 2 related viruses, human rhinovirus serotype 2 and Coxsackievirus B4, have disclosed the enzymatic active pocket of these proteases [38,39]. Because of their similarities in function and sequence, the catalytic triad EV71 2A^pro^ could be predicted as His-21, Asp39, and Cys-110. By combining the information from the structures and the predicted catalytic sites, the structure-based inhibitor design is still applicable. Moreover, antivirals targeting EV71 proteases should not only block viral protein maturation, but they may also assist in the protecting host proteins from protease degradation.

### Antivirals targeting the enterovirus RdRP complex

**Figure 2:**
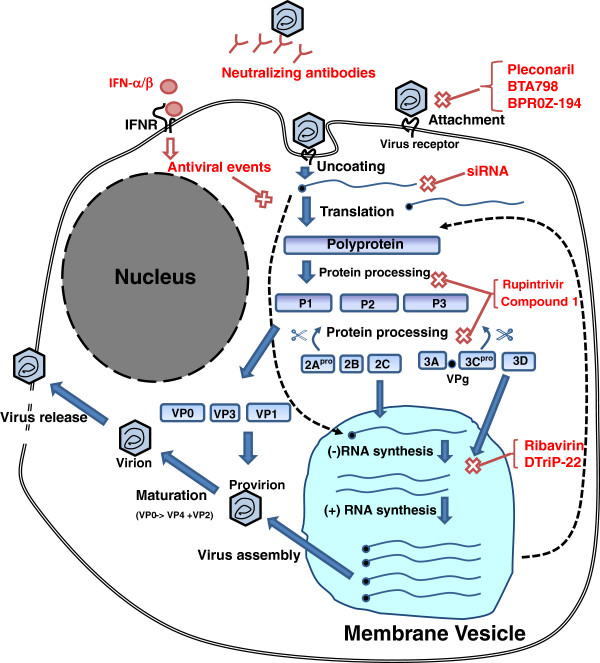
**Overview of EV71 replication and molecular mechanisms of potential inhibitors.** EV71 binds to its receptor on host cell and releases the genomic RNA into cytoplasm. Neutralizing antibodies bind to the receptor-binding capsid protein VP1 and prevent the virus-receptor interaction. VP1-binding small molecules, such as pleconaril, BTA798, and BPR0Z-194, can also interfere in virus entry. In cytoplasm, viral RNA can be targeted by virus-specific siRNAs for suppressing virus replication. A single large polyprotein is first synthesized and then cleaved by viral proteases 2A and 3C to form functional proteins. Rupintrivir and Compound 1 can inhibit 3C protease activity to prevent maturation cleveage.

Because the EV71 RNA genome is replicated by its RdRP, known as the 3D protein, targeting the 3D polymerase could be a potent strategy for specifically inhibiting EV71 replication. Nucleoside analogues, such as ribavirin (Figure 1C) and 2’-*C*-methylcystidine, have been studied most extensively as picornavirus polymerase inhibitors [40-43]. Furthermore, a non-nucleoside analogue, DTriP-22, which is a piperazine-containing pyrazolo [3,4-*d* pyrimidine derivative (Figure 1C), was identified as an anti-EV71 agent. By selecting resistant viruses, DTriP-22 was shown to inhibit viral RNA replication by targeting the EV71 3D polymerase [44]. Additionally, aurintricarboxylic acid, which was originally reported to be an inhibitor for the replicases of HCV and SARS-CoV, also exhibits the ability to inhibit EV71 3D polymerase [45-47].

### Inhibition of EV71-IRES dependent translation

Because EV71 mRNA does not have 5’ cap structure, translation is dependent on its IRES element. Numerous studies have shown that EV71 IRES-dependent translation is highly controlled by IRES-specific transacting factors (ITAFs) [48]. In addition to the ITAFs of EV71 IRES, far upstream element binding protein 2 (FBP2) was reported to negatively regulate EV71 IRES activity by competing with an ITAF named PTB [49]. By employing proteins that destructively affect EV71 IRES, the replication of EV71 can be suppressed. This concept may provide a new strategy for anti-EV71 development. For example, kaempferol, a type of flavonoid, has been shown to inhibit EV71 replication and its IRES activity by changing the composition of the ITAFs [50].

### Other small molecule antivirals targeting on EV71 replication

Enviroxime was found as an anti-viral compound against the replication of rhinovirus and poliovirus [51]. By analyzing the enviroxime-resistant mutants, the target site of enviroxime was identified on viral protein 3A [52]. The viral protein 3A and its precursor 3AB play the key roles in formation of enterovirus replication complex [53,54]. Development of anti-vrials targeting on 3A or 3AB may be a successful strategy for inhibiting EV71 replication. For example, AN-12-H5, which is a functionally enviroxime-like compound, was shown to be a novel inhibitor to block EV71 replication in vitro [55].

### The potentiality of RNA interference

RNA interference is a cellular post-transcriptional process in which gene expression is silenced in a sequence-specific manner. Based on this concept, artificially generated small, interfering RNAs (siRNAs) are widely applied to study gene function. Because siRNAs can effectively downregulate gene expression, virus sequence-specific siRNAs have been considered to be potential therapeutic agents. Several studies have shown that virus-specific siRNAs can successfully suppress the replication of human viruses, such as poliovirus, HIV-1, and HCV [56-59]. This technology has also been applied experimentally to the treatment of EV71 infection [60,61]. Scientists have employed a suckling mouse model to evaluate siRNA against EV71 in vivo, and an siRNA targeting the 3D region has been shown as a potential therapeutic approach [62].

### Modulation of host immunity and interferon treatment

Innate immunity is the host’s natural defense system against virus invasion. Production of type I interferons (IFNs), IFN-α/β, is the initial response of innate immunity and results in activation of IFN-stimulated gene expression to block viral replication. IFN-α has been used to treat HCV infection, but its application for enterovirus infection has not been established. To evaluate whether type I IFN has a therapeutic effect against EV71 infection, a recombinant murine IFN-α was administered to EV71-infected newborn mice, resulting in an increased survival rate [63]. Similar to the in vivo study, in vitro testing also demonstrated the potency of IFN-α14 in reducing EV71 replication [64]. Although it has been shown that EV71-encoded protease 3C could degrade interferon regulatory factor-9 (IRF9) which is involved in type I IFN downstream signaling, combination of IFN-α and 3C^pro^ inhibitor, rupintrivir, for EV71 treatment was considered as a strategy to combat the IFN signaling inhibition [65]. By the in vitro study, the combination treatment showed a synergistic effect against EV71 replication [65]. According to these studies, type I IFN could be considered a potent anti-EV71 treatment. Nevertheless, a recent study demonstrated that EV71 2A^pro^ could be an IFN antagonist, because it reduces the expression level of the type I IFN receptor [66], making it questionable whether type I IFN will be active against EV71 infection.

### Resistance to antiviral treatment

Because the EV71 RNA genome is synthesized by its RdRP, which does not have proofreading activity for faithfully replicating viral RNA, mutations in the newly synthesized viral genome are frequently generated during replication. Thus, EV71 variants that present antiviral resistance phenotypes could often be selected during antiviral treatment. For example, the elucidation of the mechanism of action of DTriP-22 was based on the appearance of drug-resistant mutants and whole viral genome sequencing [44]. To address the drug resistance of EV71, the practicability of combination treatments learned from HIV therapy is worth further evaluation. Ideally, molecules selected for combination therapy should act through different mechanisms. Based on two recent studies evaluating combination therapy against EV71, several combinations successfully showed a synergistic effect in inhibiting EV71, such as the combination of IFN-α and rupintrivir and the combination of rupintrivir and an analogue of pirodavir, BTA798 (Figure 1A) [65,67]. Therefore, combination therapy could be a possible strategy to combat EV71.

### Animal models for testing drugs against EV71

To verify the effectiveness of antivirals against EV71, animal studies are urgently required, in addition to in vitro experiments. Since Chumakov et al. established an experimental animal infection model, neonatal mice have been the most widely used for in vivo studies [68]. For example, in vivo studies were conducted using 1-day-old ICR mice to evaluate the protection of neutralizing antibodies against EV71 and to investigate CNS involvement after EV71 infection [22,69]. However, there are technical difficulties in handling 1-day-old pups for evaluating antiviral efficacy and toxicity. Additionally, a mouse-adapted EV71 strain was established by serial passage in mice. The mouse-adapted virus could infect 7-day-old ICR mice by oral inoculation and cause lethal CNS complications [70]. This tool therefore improved in vivo pathogenesis studies. Other than mice, cynomolgus monkeys have been used to study the neuropathogenicity of EV71 [71,72]. However, the mouse model with the mouse-adapted strain may be a relatively low-cost and effective solution for evaluating new antivirals.

### The developments of vaccine against EV71

Based on the experience in the control of poliovirus epidemic, vaccination should be a primary strategy to prevent EV71 infection in children. Although there is no commercialized effective vaccine against EV71 available until now, several researches have disclosed the availability for the vaccine against EV71. For example, the viral capsid protein VP1 was initially designed as an immunogenic candidate for subunit vaccine [73-75]. In addition to VP1 subunit vaccine, the formaldehyde-inactivated whole-virus of EV71was also widely evaluated as a vaccine candidate [76,77]. However, several obstacles need to be overcome in the EV71 vaccine development. First of all, because of the lack in adult mouse model, most of the researches applied passive immunization strategies to verify the neutralizing antibody generated from immunized dams in the protection of the newborn mice. To overcome the limitation of the mouse model, the mouse-adapted strains of EV71 or EV71-receptor-transgenic mice may be applicable to the vaccine development. Since there are many genotypes of EV71 isolates based on the VP1 sequences, the selection for reference strain could be another obstacle for vaccine production. Therefore, it is urgent to establish the international collaboration for sharing the virology and epidemiological information. Nevertheless, a phase I clinical trial in human has been conducted to evaluate the safety and immunogenicity of a newly developed inactivated EV71 vaccine in China [78].

## Summary

Over the past 2 decades, eradication efforts have substantially reduced the number of poliomyelitis cases worldwide. Unfortunately, reemerging EV71 infection has become another challenge for public health, especially in the Asia-Pacific region. Unlike poliomyelitis, an effective EV71 vaccine is still not available to provide immunity in children. Therefore, development of anti-EV71 agents has become an urgent issue to relive distress in epidemic areas. Based on the replication characteristics of picornaviruses, several strategies have been developed for designing antivirals against EV71, as summarized in Figure 2. Certain antivirals originally developed against human rhinoviruses have been tested against EV71 because of the similarity in viral replication mechanisms. Because an experimental mouse model has been established, candidate anti-EV71 drugs can be further evaluated to assess therapeutic efficacy.

## Competing interests

The authors declare that they have no competing interests.

## Authors’ contributions

RLK wrote the manuscript. SRS participated in its organization and coordination and helped to draft the manuscript. Both authors read and approved the final manuscript.
